# DISCUSSION OF AGE-FRIENDLY UNIVERSITY PRINCIPLES WITH OLDER LEARNERS

**DOI:** 10.1093/geroni/igac059.1711

**Published:** 2022-12-20

**Authors:** Emiko Takagi, Madeleine Marroquin-Serrano

**Affiliations:** San Francisco State University, San Francisco, California, United States; San Francisco State University, San Francisco, California, United States

## Abstract

The purpose of this project was to study how older learners think a university campus currently meets the 10 Age-Friendly University principles and what they see as potential steps to create an age-friendly campus community. Online focus group interviews were conducted with 17 members of Osher Lifelong Learning Institute at San Francisco State University in 2020. The participants were 60 years or older, and the majority were female and non-Hispanic white. The study participants received information about the 10 Age-Friendly University principles presented by the Age-Friendly University Global Network and were asked to discuss their thoughts about how the university satisfies these principles. The interview recordings were transcribed for the thematic analysis of qualitative data. The analysis yielded three themes. The first theme described the diversity of older adults’ learning needs and desires that the university must recognize and accommodate. The second theme represented older adults’ sense of optimism and anxiety about intergenerational learning. The third theme highlighted the challenges older adults tend to experience in accessing information, educational programs, and/or university facilities. The interviews with older learners were found valuable and indispensable in the work of Age-Friendly University assessments. The presentation is focused on the discussion regarding how older adults’ voices can be incorporated in the assessments and ways in which higher education institutions should combat ageism on and off campus as part of work to address the issues identified in this study.

